# The Iron Response of *Mycobacterium tuberculosis* and Its Implications for Tuberculosis Pathogenesis and Novel Therapeutics

**DOI:** 10.3389/fcimb.2022.876667

**Published:** 2022-05-11

**Authors:** G. Marcela Rodriguez, Nishant Sharma, Ashis Biswas, Nevadita Sharma

**Affiliations:** The Public Health Research Institute, Department of Medicine, Rutgers University, New Jersey Medical School, Newark, NJ, United States

**Keywords:** *M. tuberculosis*, iron-limitation, IdeR, ferritin, extracellular vesicles, iron-response

## Abstract

Most pathogenic bacteria require iron for growth. However, this metal is not freely available in the mammalian host. Due to its poor solubility and propensity to catalyze the generation of reactive oxygen species, host iron is kept in solution bound to specialized iron binding proteins. Access to iron is an important factor in the outcome of bacterial infections; iron limitation frequently induces virulence and drives pathogenic interactions with host cells. Here, we review the response of *Mycobacterium tuberculosis* to changes in iron availability, the relevance of this response to TB pathogenesis, and its potential for the design of new therapeutic interventions.

## Introduction

Iron is an essential micronutrient for most living organisms. It undergoes reversible changes in its oxidation state, oscillating between the oxidized ferric (Fe^3+^) and the reduced ferrous (Fe^2+^) forms. Depending on the local ligand environment, iron-containing compounds exhibit a wide range of oxidation-reduction potentials making this metal a multipurpose biocatalyst and electro-carrier. Indeed, as mono-or binuclear species, in heme groups or iron-sulfur clusters [FeS], iron is incorporated into proteins that mediate vital cellular functions, including energy generation, metabolism, oxygen transport, gene regulation, oxidative stress defense, and DNA biosynthesis (Sanchez et al., 2017).

Before oxygenic photosynthesis, iron was abundant in its soluble, Fe^2+^ form and incorporated into a variety of enzyme cofactors. However, with the introduction of oxygen into the atmosphere, iron became poorly available and potentially toxic. In oxygenic environments, Fe^+3^ forms insoluble ferric hydroxides and promotes the generation of deleterious reactive oxygen species (ROS) thorough the Fenton reaction (Fenton, 1894; Imlay et al., 1988; Koppenol and Hider, 2019). The mammalian host keeps iron in solution bound to proteins such as transferrin (in plasma), lactoferrin (in secretory fluids), and ferritin (intracellularly). During the acute phase response to infection, iron-binding proteins are upregulated together with proteins that efflux iron from intracellular microbial compartments (NRAMP1) or bind free heme (hemopexin) and hemoglobin (haptoglobin), further reducing iron available to invading microorganisms (Weinberg, 1999). This concerted effort mounted by the host to actively restrict iron availability constitutes the hallmark of nutritional immunity (Weinberg, 1984).

Tuberculosis (TB), caused by *Mycobacterium tuberculosis* (Mtb), is a leading cause of death from a single infectious agent. However, tools to effectively prevent and control TB remain scarce. BCG (Bacillus Calmette-Guerin), the only approved antitubercular vaccine protects against disseminated TB in infants, but is inefficient against adult pulmonary TB (Andersen and Doherty, 2005). Four different antibiotics taken for four months are needed to treat TB. This complex antibiotic regimen contributes to poor adherence and the rise of drug resistant Mtb strains. It is estimated that one-third of the world’s population has been exposed to Mtb and might harbor bacteria that are not multiplying but remain viable causing chronic, latent TB infection (LTBI). LTBI is asymptomatic and not transmissible but nonetheless, highly problematic because it is difficult to diagnose and treat. Moreover, when immune control is compromised, LTBI can reactivate generating new acute TB cases (Lillebaek et al., 2002).

Mtb enters a new host *via* inhalation of fine aerosol droplets containing just a few bacilli. In the lung, Mtb creates an optimal niche for replication within alveolar macrophages. But it can also disseminate and replicate in multiple organs. Bacterial proliferation stimulates a pro-inflammatory response resulting in the recruitment of more macrophages and other immune cells, forming a granuloma. As adaptive immunity develops, the granuloma can restrict bacterial growth. However, cells in the granuloma can undergo necrosis, forming a necrotic core where Mtb is found extracellularly (Ramakrishnan, 2012). At these various stages of infection, Mtb experiences distinct iron conditions to which it must acclimate.

**Figure 1:**
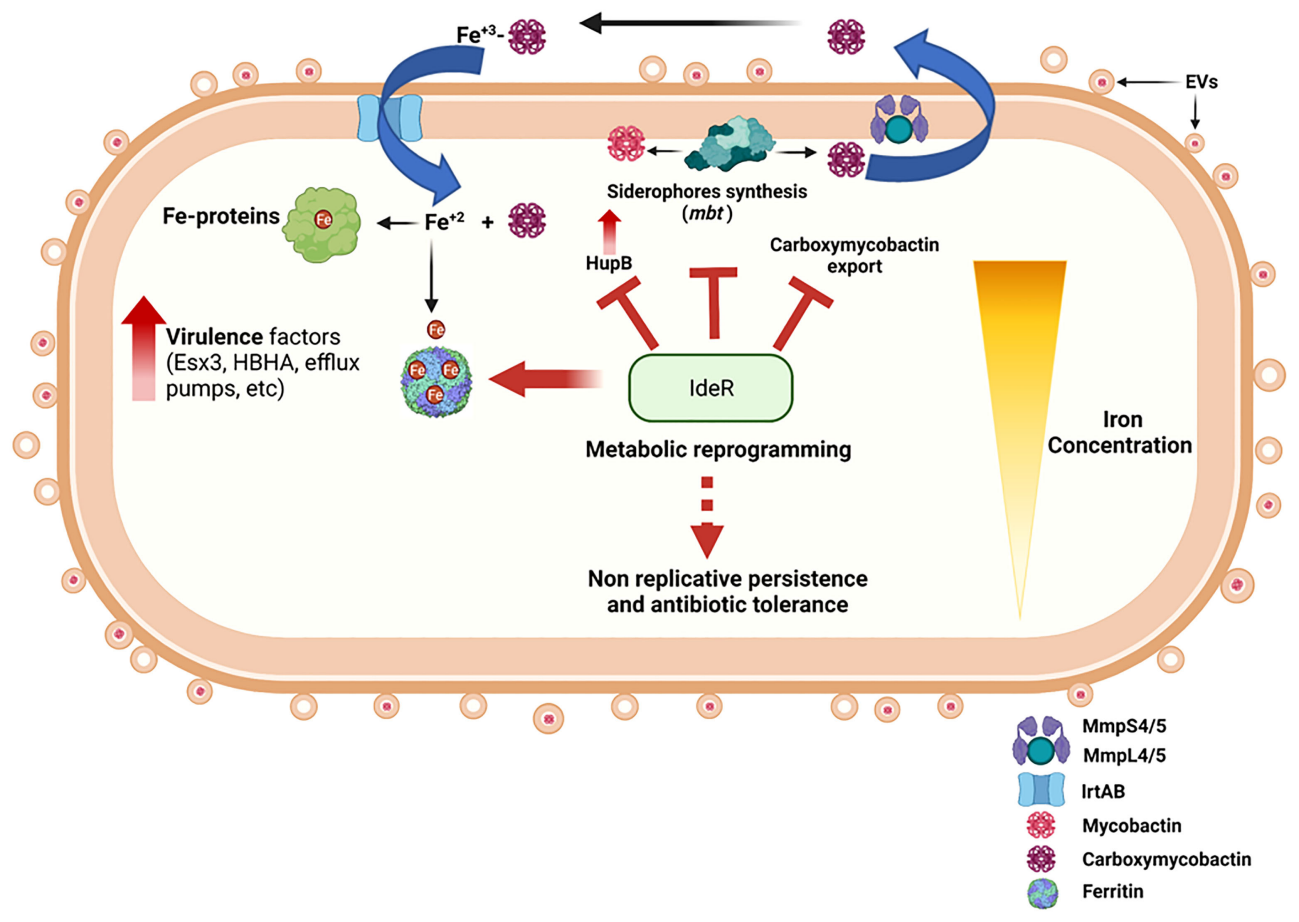
Summary diagram of the Mtb response to iron availability. Iron limited Mtb upregulates expression of genes encoding siderophore synthesis (*mbt*), export (MmpL4/5-MmpS4/5), and import (IrtAB). Assimilated iron is incorporated into metalloproteins and stored in ferritins. Iron deficient Mtb also upregulates pathogenicity factors that facilitate immune evasion and proliferation, modifies its cell surface, and augments EVs secretion. Prolonged iron deprivation induces a strong iron sparing response and metabolic rewiring that enables long term persistence without replication and leads to phenotypic antibiotic resistance. These changes are fully reversible by restoring iron availability. Iron sufficient Mtb engages IdeR to control intracellular iron levels *via* repression of iron uptake and stimulation of iron storage, thus preventing iron dysregulation and oxidative stress that renders this pathogen highly vulnerable to host antimicrobial factors and antibiotic drugs. The figure was created with BioRender (BioRender.com.).

This review focuses on the intricate response of Mtb to changes in iron availability. Depending on accessibility to iron, this response ranges from increased iron acquisition, virulence, and growth to quiescence and long-term persistence (**Figure 1**). We highlight how the physiological response of Mtb to diverse iron conditions can influence TB pathogenesis and offer new opportunities for therapeutic intervention.

## Upregulation of Iron Acquisition and Control of Intracellular Iron

Iron is highly abundant in the human host. But it is not readily accessible to infectious agents. It is always bound to iron-binding proteins or coordinated into the cofactor, heme. To overcome iron limitation, Mtb produces siderophores, namely the lipophilic, membrane-bound mycobactin, and the water-soluble, secreted carboxymycobactin (Hall and Ratledge, 1982; Barclay et al., 1985; Gobin et al., 1995; Lane et al., 1995). Additionally, in a siderophore-independent manner, Mtb can utilize heme as iron source (Jones and Neiderweis, 2011; Tullius et al., 2011; Mitra et al., 2019). Carboxymycobactin and mycobactin share a common core structure composed of a 2-hydroxyphenyl-oxazoline moiety, a β-hydroxy carbonyl motif and two N^ε^- (hydroxy) lysin residues (Gobin et al., 1999). Mycobactin has a long alkyl chain substitution that makes this molecule highly hydrophobic and restricts it to the cell envelope (Ratledge et al., 1982). Mycobactin and carboxymycobactin have an extremely high affinity for Fe^3+^ and can withdraw it from insoluble hydroxides and iron-binding proteins (Gobin and Horwitz, 1996). Accordingly, Mtb requires siderophore synthesis to proliferate in macrophages and mice (De Voss et al., 2000; Reddy et al., 2013).

While carboxymycobactin is exported *via* the inner-membrane RND transporters MmpL4 and MmpL5 in association with the periplasmic adaptor proteins MmpS4 and MmpS5 (Wells et al., 2013), mycobactin is exported as a component of extracellular membrane vesicles (Prados-Rosales et al., 2014). Carboxymycobactin can transfer chelated Fe^3+^ to mycobactin on the cell surface by a process that requires the multifunctional protein HupB. HupB is primarily a nucleoid-associated protein that activates the transcription of siderophore synthesis genes (*mbt*). But surprisingly, HupB is also found on the cell surface, where it can bind carboxymycobactin. HupB has been postulated to serve as Fe^3+^-carboxymycobactin receptor and direct transfer of iron from carboxymycobactin to mycobactin (Gobin and Horwitz, 1996; Choudhury et al., 2021). Iron bound to carboxymycobactin can also be imported into the cell independently of mycobactin, *via* the iron-regulated ABC transporter, IrtAB (Rodriguez and Smith, 2006; Rodriguez et al., 2008; Ryndak et al., 2010; Arnold et al., 2020). IrtAB is an inner membrane heterodimer and a unique transporter that couples Fe^3+^-carboxymycobactin import and iron assimilation *via* the cytosolic amino-terminal domain of IrtA (Ryndak et al., 2010). This domain functions as a flavin reductase, reducing Fe^3+^ to Fe^2+^, effectively dissociating Fe^+2^ from imported carboxymycobactin and from membrane associated mycobactin for subsequent incorporation into iron proteins (Ryndak et al., 2010; Arnold et al., 2020). Deletion of *irtAB* significantly impairs Mtb proliferation in macrophages and in mice (Rodriguez and Smith, 2006). The recent characterization of the three-dimensional structure of IrtAB is an important step towards developing therapeutics that target iron uptake in Mtb (Gold et al., 2001; Arnold et al., 2020).

Fe^3+^-siderophore uptake also requires the type VII Esx-3 secretion system (Siegrist et al., 2009; Serafini et al., 2013). However, the precise function of the Esx-3 proteins in iron uptake remains unclear. The Esx-3 system is also necessary for Mtb virulence and therefore, an attractive drug target (Tufariello et al., 2016).

Once deferrated, carboxymycobactin is recycled and exported back. Failure to export carboxymycobactin is detrimental to Mtb *in vitro* and *in vivo*, supporting siderophore export proteins as promising anti-Mtb drug targets (Jones et al., 2014).

Carboxymycobactin and mycobactin are both potent iron chelators. However, carboxymycobactin molecules are susceptible to lipocalin, a protein induced under inflammatory conditions that sequesters carboxymycobactin and thereby interferes with iron acquisition (Goetz et al., 2002). Accordingly, lipocalin deficient mice are highly susceptible to tuberculosis infection and exhibit increased bacterial burden, particularly in the alveolar epithelium (Saiga et al., 2008). In contrast, mycobactin packed into extracellular membrane vesicles might be less exposed to lipocalin and consequently more effective in delivering iron to Mtb in the context of inflammation. Thus, we postulate that the need to sequester iron in biochemically diverse host environments justifies the synthesis of two types of siderophores by Mtb.

To prevent toxicity derived from excess iron and maintain iron homeostasis, Mtb stores iron in bacterioferritin (BfrA) and ferritin (BfrB) (Gupta et al., 2009; Khare et al., 2011; Reddy et al., 2012). Ferritins characteristically exist as spherical macromolecular assemblies of 24 identical subunits. BfrB has a higher iron storage capacity than BfrA. BfrA can take up 4500 molecules of iron/protein whereas BfrB could take up to 6000 iron molecules/protein (Khare et al., 2017). The synthesis of BfrB is induced in high iron conditions whereas BfrA is synthesized in low and high iron medium. BfrB is the main storage compartment for excess iron. Mtb with deleted *bfrB* accumulates reactive iron, exhibits iron-dependent hypersensitivity to ROS and antibiotics, and survives poorly in the lungs of mice, particularly during the chronic phase of infection (Pandey and Rodriguez, 2012; Reddy et al., 2012). Notably, immunization of mice with a *bfrB* mutant of Mtb confers the same level of protection against virulent Mtb as compared to BCG vaccination (Subbian et al., 2014). BfrA on the other hand, is a heme-containing protein, that contributes to maintaining iron homeostasis during iron limitation (Khare et al., 2017). In addition, BfrA exhibits catalase and DNA protection activity; it associates with DNA and protects it from Fe^2+^/H_2_O_2_ -induced oxidative damage (Mohanty et al., 2019). Although these findings highlight the potential of targeting mycobacterial ferritins to potentiate host and antibiotic-mediated killing of Mtb, direct inhibition of ferritins is challenging due to their compact molecular assembly and their homology to host ferritin. Alternatively, we postulate that targeting upregulation of ferritin in response to high iron conditions might lead to accumulation of oxygen radicals *via* Fenton chemistry and synergize with existing antitubercular antibiotics.

Mtb maintains cellular iron homeostasis by tightly regulating iron uptake, utilization, and storage. Siderophore synthesis is activated by the nuclei-associated protein HupB (Pandey et al., 2014) and repressed by the metal-dependent regulator IdeR (Dussurget et al., 1996; Pohl et al., 1999; Gold et al., 2001; Rodriguez et al., 2002). IdeR binds Fe^2+^ and DNA at a specific sequence present in the promoters of siderophore synthesis, export and import genes, as well as upstream of *hupB*, *bfrA*, and *bfrB* genes (Chou et al., 2004; Marcos-Torres et al., 2021). According to the location of the IdeR binding site (iron box), IdeR functions as a repressor or activator of gene transcription. Generally, iron uptake genes have a single iron box that overlaps the promoter or transcriptional start site. Therefore, binding of IdeR to this sequence represses their transcription. In contrast, IdeR binding sites on *bfrA* and *bfrB* are located further upstream of the promoter region (Gold et al., 2001). IdeR activates *bfrB* transcription by antagonizing the histone-like protein Lsr2, which decorates the DNA region upstream of *bfrB* impeding its transcription. In complex with iron, IdeR binds to four tandem iron boxes located upstream of *bfrB*, likely displacing Lsr2 and aiding recruitment of RNA polymerase to the promoter (Kurthkoti et al., 2015). A strain unable to synthesize IdeR fails to establish infection in mice, indicating that controlling the response to iron is essential *in vivo* and validating IdeR as a promising drug target against Mtb (Pandey and Rodriguez, 2013). Despite the importance of IdeR as a drug target, only few virtual screenings for IdeR inhibitors have been reported so far (Rohilla et al., 2017; Kwofie et al., 2019). Transcriptional factors are notably difficult to target since protein-protein or protein-DNA interactions must be disrupted. However, since metal binding is needed for IdeR dimerization and DNA binding, blocking metal binding may be an effective way to inhibit IdeR function (Wisedchaisri et al., 2007). High throughput screenings for natural or synthetic IdeR inhibitors are needed to capitalize on Mtb’s marked susceptibility to iron dysregulation.

## Upregulation of Virulence-Associated Genes

Iron limitation stimulates many bacterial pathogens to synthesize and release virulence factors that directly or indirectly increase iron bioavailability. For instance, enhanced production of hemolysins, cytotoxins, phospholipases, and proteases allows pathogens to release intracellular iron *via* cell lysis (Dorman et al., 1990; Tai et al., 1990; Karjalainen et al., 1991; Litwin and Calderwood, 1993; Garcia et al., 2003). More subtly, adhesins and motility proteins might provide access to new iron-supportive microenvironments. Other factors enable pathogens to highjack iron trafficking pathways of the host (Keenan and Allardyce, 2000).

Transcriptional profiling, gene deletion, and animal infection studies indicate that Mtb reacting to iron limitation simultaneously upregulates iron acquisition mechanisms and diverse virulence associated factors (Rodriguez et al., 2002) (Kurthkoti et al., 2017). For instance, iron limitation induces *pks10* (Sirakova et al., 2003) and *mmpL8* (Converse et al., 2003) encoding two proteins necessary for synthesis of the virulence associated cell envelope lipids dimycoserosyl phthiocerol (DIM) and sulfolipid-1 respectively. The phospholipase gene *plcA* and the cholesterol oxidase gene *choD*, which are required for virulence in macrophages are also induced by iron restriction (Raynaud et al., 2002; Brzostek et al., 2007). In addition, iron-deficient Mtb upregulates the mammalian cell entry protein-encoding gene mce3C (Yuan et al., 1998; Senaratne et al., 2008; Garces et al., 2010), the adhesin HbHA, and the genes encoding the virulence required ESX-1 secretion associated proteins, EspA and EspC (Garces et al., 2010). Furthermore, low iron signals induction of genes encoding regulatory proteins that control the expression of many genes and are functionally linked to Mtb survival *in vivo*, such as HspX, MprAB, SigF, and WhiB6 (Yuan et al., 1998; Zahrt and Deretic, 2001; Geiman et al., 2004; Chen et al., 2016).

Notably, Mtb evolved the ability to infect and survive in macrophages, cells that are central to host iron homeostasis. Macrophages are responsible for recycling iron from hemoglobin in senescent red blood cells (the largest iron pool in the host) and exporting it to satisfy systemic iron demand (Armstrong and Hart., 1975; Ganz, 2012). Mtb infecting macrophages inhibits phagosome-lysosome fusion while allowing phagosome interactions with early endosomes containing internalized transferrin, a reliable iron source accessible to Mtb *via* siderophores (Russell, 1996; Sturgill-Koszycki et al., 1996; Wagner et al., 2005; Clemens and Horwitz, 1996; Olakanmi et al., 2002). Therefore, it is not surprising that Mtb sensing iron deficiency upregulates phagosome maturation arresting factors such as the Esx-3 secretion apparatus and the protein kinase, PknG (Mehra et al., 2013; Cambier et al., 2014; Rodriguez et al., 2002).

During inflammation, macrophages are “instructed” by hepcidin, the principal regulator of systemic iron, to retain this metal intracellularly effectively restricting iron available to extracellular invaders (Drakesmith and Prentice, 2012). High macrophage iron stores are indeed associated with an increased risk of developing tuberculosis, while IFN-γ activated macrophages that downregulate iron uptake and reduce iron storage are less hospitable to Mtb and can eliminate it (Byrd and Horwitz, 1989). Suggestive of mycobacteria exploiting the macrophage for iron, infected macrophages show increased hepcidin secretion and accumulate intracellular iron (Abreu et al., 2020). Interestingly, Mtb infection can induce macrophage ferroptosis, a form of lytic cell-death resulting from iron dependent lipid peroxidation and membrane damage (Amaral et al., 2019). We think ferroptosis might be mechanistically linked to Mtb’s efforts to increase iron available in the macrophage. Future studies are needed to validate this assumption. In the mature granuloma, Mtb promotes inflammation and cell necrosis that combined with the ability to utilize iron from heme and hemoglobin likely contribute to extracellular Mtb multiplication. A better understanding of the Mtb-macrophage interactions driven by iron limitation will likely uncover additional strategies used by Mtb to manipulate the macrophage and the immune system to secure essential iron.

## Cell Surface Modification

Accumulated evidence from genetic and biochemical analyses indicates that iron restricted Mtb alters its cell envelope. Phenotypically, mycobacteria cultured in low iron medium exhibit increased aggregation, enhanced permeability and membrane fluidity associated with hypersensitivity to membrane perturbing agents and antibiotics (Pal et al., 2015). Iron-deprived mycobacteria reduce expression of MmpL3 (Pal et al., 2019), a lipid transporter responsible for translocating trehalose monomycolate to the periplasm for the biosynthesis of trehalose dymicolates (TDM) and mycolyl arabinogalactan peptidoglycan, which are basic structural components of the mycobacterial cell envelope (Xu et al., 2017). In addition, iron-deprived mycobacteria exhibit decreased mycolic acid content suggestive of reduced outer membrane biogenesis (Bacon et al., 2007; Xu et al., 2017). Furthermore, a link between iron limitation and increased phospholipid catabolism was postulated based on lipidomic analysis and cell-wall lipid profiling that showed phospholipid reduction in iron deficient Mtb, a significant increase in triacylglycerols, and the presence of a novel wax ester in iron limited Mtb (Madigan et al., 2015). These observations and the significant number of uncharacterized iron regulated genes predicted to be involved in lipid metabolism support the concept that Mtb alters its cell envelope in response to iron limitation (Rodriguez et al., 2002; Kurthkoti et al., 2017). Controlled cell surface modification may facilitate iron uptake and expose biomolecules that enable novel interactions with host cells. Comprehensive studies that further characterize structural and functional changes in the cell envelope of iron limited Mtb might reveal new adaptations to the host environment. Additionally, knowledge about the molecular mechanisms used by Mtb to control cell envelope permeability with respect to iron can be harnessed to enhance antibiotic cell entry and effectiveness.

## Increase Release of Extracellular Vesicles

Iron limitation stimulates the release of mycobacterial extracellular vesicles (EVs) (Prados-Rosales et al., 2014). EVs are spherical, membrane-bound nanoparticles (60-300 nm in diameter) released by live cells to communicate with other cells in their environment. EVs carry bioactive molecules through the body, signaling distant tissues and triggering systemic responses. Pathogenic bacteria frequently secrete virulence factors concentrated and protected within EVs (Kuehn and Kesty, 2005). Mycobacteria produce EVs that originate at the plasma membrane and contain immunologically active lipids and proteins, including numerous TLR2 ligands (Prados-Rosales et al., 2011). Emerging evidence indicates that bacteria derived EVs might contribute to TB pathogenesis. EVs released by Mtb in culture or during macrophage infection influence cellular immune responses (Athman et al., 2015; Athman et al., 2017) and when injected into mice, mycobacterial EVs stimulate inflammation and interfere with the control of a subsequent Mtb infection (Prados-Rosales et al., 2011). EVs produced by iron limited Mtb may also aid in iron acquisition. They are loaded with mycobactin and can support the growth of iron deficient mycobacteria *in vitro*. As mycobactin can accept Fe^+3^ from carboxymycobactin, mycobactin packed in EVs is likely a mix of Fe^+3^-mycobactin and apo-mycobactin capable of chelating environmental iron (Prados-Rosales et al., 2014).

We demonstrated that EV biogenesis in Mtb is dependent on the function of the dynamin-like proteins IniA and IniC. Accordingly, the *iniBAC* operon is induced during iron limitation (Gupta et al., 2020). Additionally, we propose that the cell envelope remodeling implemented by iron limited Mtb might be linked to increased EV production; conceivably, a more porous cell envelope could facilitate vesicle export. Further studies are needed to elucidate the relationship between cell envelope alteration and EV secretion in Mtb. Nonetheless, the current information indicates that EV-mediated secretion is yet, another pathogenicity associated function augmented in Mtb responding to iron restriction.

## Metabolic Reprogramming

Without iron, Mtb downregulates anabolic functions and stops growth. Iron deficiency triggers intense metabolic adjustment broadly influenced by an effective iron-sparing response. This response is characterized by repression of dispensable iron containing proteins and prioritization of scarce iron for life sustaining activities. Specifically, the transcriptional profile of iron-deprived Mtb indicates the prioritization of [FeS] assembly over heme biosynthesis and the maintenance of iron proteins involved in electron transfer (Cyp138, FprB, RubA, and RubB) and NAD synthesis (NadA); and the [FeS]-containing regulators WhiB6 and WhiB7 (Kurthkoti et al., 2017). Metabolic and transcriptomic analyses of iron starved Mtb indicate reduced amino acid biosynthesis. Energy metabolism including the tricarboxylic acid (TCA) cycle and oxidative phosphorylation are also downregulated. This is expected given the number of [FeS]/hemeproteins involved in these pathways. Concomitantly, the expression of *atp* genes (encoding F_1_F_0_ ATP synthase) decreases, indicating reduced respiration associated ATP synthesis (Kurthkoti et al., 2017). We postulated that like *Lactobacillus* and *Borrelia*, two unique bacterial species that do not depend on iron and lack a functional TCA cycle and oxidative phosphorylation, iron starved Mtb might generate ATP *via* substrate phosphorylation. Also, *Lactobacillus sp* and *Borrelia sp* depend on pyruvate conversion to lactate to regenerate NAD+ required for continued glycolysis. Metabolic detection of lactate suggests that Fe-starved Mtb also performs homolactic fermentation. Additional studies are needed to identify metabolic adaptations that support iron starved Mtb and can be exploited as novel therapeutic targets to prevent chronic Mtb infection.

## Iron-Starved M. Tuberculosis Enters a Quiescent State and Becomes Tolerant to Antibiotics

Characterization of Mtb cultures adapted to iron-starvation revealed a multi-phased response that included cell death, differential cultivability (DC), and non-replicative persistence (Kurthkoti et al., 2017). DC refers to the ability of bacterial cells to grow in liquid but not in a solid medium. This property is also observed in Mtb under intense stress and in bacteria present in sputum samples from TB patients before treatment (Mukamolova et al., 2010). The DC phenotype is troubling because it potentially decreases the sensitivity of culture-based diagnostics and treatment efficacy assessments, which are still broadly used in low-resource settings.

Most Mtb cells adapt to complete iron deprivation by entering a non-replicative, persistent state fully reversible by iron (Kurthkoti et al., 2017). Iron starved Mtb ceases to replicate and becomes tolerant to several antibiotics, including the first-line TB drug isoniazid (INH), also used in latent TB reactivation preventive therapy (Kurthkoti et al., 2017). Although enhanced tolerance to antibiotics in non-growing cells can be simply explained by reduced abundance of the drug target, the response to lack of iron may specifically contribute to antibiotic tolerance. For instance, INH is a prodrug activated by catalase (KatG), a heme enzyme downregulated in response to iron-deprivation (Chouchane et al., 2000; Chouchane et al., 2003). Therefore, reduced INH activation may contribute to increased drug tolerance in iron starved Mtb. In addition, inhibition of the electron transport chain due to Fe-deficiency likely mediates enhanced tolerance to antibiotics such as aminoglycosides which require proton motive force for internalization into bacterial cell (Davis, 1987). Furthermore, the genes encoding recognized intrinsic antibiotic resistance-enhancers such as WhiB6 (Chen et al., 2016) and WhiB7(Ramon-Garcia et al., 2013) and the fluoroquinolone efflux pump encoded by Rv2688c (Pasca et al., 2004) are also induced in response to iron deficiency, supporting an active role of the iron response in antibiotic tolerance (Kurthkoti et al., 2017).

Proteomic analysis of micro dissected lung granulomas obtained from individuals with extensive drug-resistant TB showed a high concentration of iron restricting host proteins in the necrotic center of advanced granulomas, which suggests the bacteria present in this microenvironment experience intense iron deprivation (Kurthkoti et al., 2017). Therefore, the capacity of Mtb to survive without iron in the face of a robust nutritional immunity might enable it to persist causing a chronic infection. This concept is compatible with reports of latent TB reactivation in individuals treated for anemia with iron supplements (Trousseau, 1872; Murray et al., 1978). Considering the data and the high prevalence of anemia worldwide, caution is recommended when implementing iron supplementation programs particularly in TB endemic areas.

## Conclusions and Perspectives

It has become clear in recent years that the Mtb response to iron limitation encompasses multiple aspects of bacterial cell biology and physiology that influence host-pathogen interactions and potentially shape the outcome of TB infection. Studies of this response have offered promising targets for intervention particularly IdeR and BfrB, whose inhibition leads to lethal iron intoxication and increased susceptibility to antibiotics. Inhibiting siderophore recycling is another promising way of killing Mtb. In contrast, targeting iron acquisition, unless done very early after infection, might trigger a persistent state characterized by antibiotic tolerance. Genetic evidence suggests that utilization of iron stored in ferritin enables long-term survival of Mtb without environmental iron (Kurthkoti et al., 2017). Therefore, targeting BfrB might also effectively preclude chronic TB infection. Implementing drug screenings using iron starved Mtb may lead to new compounds effective against Mtb surviving iron starvation in the host and causing latent TB, a form of TB that continues to affect over one billion people worldwide.

## Author Contributions

GMR, NeS, AB, and NiS wrote the manuscript. All authors contributed to the article and approved the submitted version.

## Funding

AB is supported by NIH AI159055. GMR, NeS, and NiS are supported by AI162821.

## Conflict of Interest

The authors declare that the research was conducted in the absence of any commercial or financial relationships that could be construed as a potential conflict of interest.

## Publisher’s Note

All claims expressed in this article are solely those of the authors and do not necessarily represent those of their affiliated organizations, or those of the publisher, the editors and the reviewers. Any product that may be evaluated in this article, or claim that may be made by its manufacturer, is not guaranteed or endorsed by the publisher.
